# Residential Environment, Depressive Symptoms, and Anxiety Symptoms Among Community-Living Older Adults

**DOI:** 10.1093/geroni/igab046.2370

**Published:** 2021-12-17

**Authors:** Ethan Siu Leung Cheung, Jinyu Liu

**Affiliations:** 1 Columbia University, New York, New York, United States; 2 Columbia University, Columbia University, New York, United States

## Abstract

Past literature has suggested significant relationships between neighborhood environment and mental health of older adults. However, the effect of residential environments is underexplored. The present study aims to study: (Q1) how residential built environments are associated with depressive and anxiety symptoms among community-living older adults, and (Q2) whether the associations of their physical and cognitive health status with mental health vary by residential environments. We analyzed data from Round 9 of National Health and Aging Trends Study. Residential environments were indicated by home despair, cluttered home, and existence of entrance ramp. Covariates included age, gender, race, living arrangement, ADL limitations, physical capacity, and cognitive status. The logistic regression results show that higher levels of clutter at home and the lack of entrance ramp were significantly associated with more depressive symptoms and that levels of clutter were positively associated with anxiety symptoms. Residential environments significantly moderated the association between physical health and mental health. With similar physical capacity, older adults with higher levels of home despair and clutter had more depressive and anxiety symptoms. Older adults who had more cluttered home reported significantly higher levels of anxiety than those who had similar ADL limitations, but lived in a less cluttered housing environment. However, we didn’t find any moderating effect of residential environments on cognitive impairment and mental health. Our findings promote the necessity for practitioners and policymakers to consider the effect of residential environments on mental health among both physically healthy and impaired older adults in the United States.

